# Factors associated with treatment-emergent headache occurrence following RTMS to non-motor areas and the impact on treatment outcomes: a retrospective study

**DOI:** 10.3389/fpsyt.2026.1814081

**Published:** 2026-05-25

**Authors:** Gauri Gusain, Thomas E. Valles, Margaret G. Distler, Evan H. Einstein, Nathaniel D. Ginder, Ralph J. Koek, David E. Krantz, Michael K. Leuchter, Hanadi A. Oughli, Aaron Slan, Thomas Strouse, Scott A. Wilke, Alexander S. Young, Andrew F. Leuchter, Juliana Corlier

**Affiliations:** 1 TMS Clinical and Research Service, Neuromodulation Division, Semel Institute for Neuroscience and Human Behavior at University of California, Los Angeles (UCLA), Los Angeles, CA, United States; 2 Department of Psychiatry & Biobehavioral Sciences, David Geffen School of Medicine at UCLA, Los Angeles, CA, United States

**Keywords:** depression, Transcranial Magnetic Stimulation (TMS), neurostimulation, side effects, headaches

## Abstract

**Background:**

Repetitive Transcranial Magnetic Stimulation (rTMS) is an FDA-approved, non-invasive treatment for Major Depressive Disorder (MDD). rTMS has a favorable safety profile, however, headaches are a common side effect. It is unclear which patients may be at greatest risk for headache, which device parameters might be associated with headache, and whether occurrence of headaches during rTMS affects eventual treatment outcome.

**Methods:**

This retrospective study analyzed clinical records from 938 patients who received rTMS treatment between 2017 and 2023. Patients were categorized based on whether they reported treatment-emergent headaches during the first 15 sessions. We assessed associations between headache occurrence and stimulation parameters (motor threshold, stimulation intensity), as well as patient characteristics (age, gender, baseline depression severity). Depression outcomes were measured using the Inventory of Depressive Symptomatology IDS.

**Results:**

Treatment-emergent headaches occurred more frequently in younger patients (p = 0.03) and women (p = 0.001), with no differences in headache occurrence observed across stimulation parameters. Patients who reported headaches had higher baseline depression scores (p = 0.0008) and maintained slightly elevated symptoms throughout treatment. Headache occurrence did not significantly affect overall clinical response rates but was associated with improved treatment retention.

**Conclusions:**

Treatment-emergent headaches during rTMS appear more closely linked to patient specific characteristics, such as age, gender, and depression severity rather than stimulation parameters. While the occurrence of headaches is common, they had no impact on clinical outcomes and can improve retention if correctly managed. We recommend providing individualized patient support during the early phase of treatment to improve adherence and enhance patient experience.

## 1 Introduction

Repetitive Transcranial Magnetic Stimulation (rTMS) is a non-invasive neuromodulation technique with robust demonstrated efficacy for treating Major Depression Disorder (MDD) (1, 2). While rTMS is considered a low-risk procedure, possible side effects include a risk of a seizure [a rare occurrence with less than 1 in 30,000 treatment sessions and less than 1 in 1,000 patient exposures (3)] and more commonly fatigue, anxiety, agitation, headaches, or scalp discomfort (4, 5).

Headaches in particular, were reported to affect patients’ overall clinical experience and possibly compromise clinical treatment adherence (6, 7). Previous studies indicate that headaches are a relatively common side effect of rTMS, typically ranging from mild to moderate severity, and may discourage some patients from persisting with the therapy (8). Recognizing the factors underlying the occurrence of headaches as a side effect of rTMS treatment is essential for optimizing rTMS protocols to reduce the patient’s burden and enhance overall clinical benefit.

Parameters such as coil type, coil placement, stimulator type, and stimulation frequency can all affect the patients’ rTMS experience and comfort. For example, stimulation intensity is a primary factor in the risk of discomfort and seizures (5, 9, 10). Stimulator type characteristics (pulse width and waveform, cooling features, and stimulation frequency can also indirectly affect discomfort (11). While the safety profiles and headache occurrence are comparable between the two most common protocols, intermittent theta burst stimulation (iTBS) and 10 Hz, one study reported that self-rated intensity of pain was higher in the iTBS group (12). Together, these findings suggest that while the overall safety characteristics are similar between most common machines and protocols, certain stimulation parameters can have differential influence on the patient tolerance and side effects incidents.

Though rTMS induces headaches in some patients, it can also be used to treat and prevent headaches in others. For example, a single-pulse TMS (sTMS) protocol has received FDA approval for both the acute and preventive treatment of migraine with aura (13). Clinical studies have demonstrated its efficacy in reducing headache frequency and severity, offering a non-invasive option for patients who may not tolerate or respond to pharmacological therapies (14). Additionally, both low and high-frequency repetitive transcranial magnetic stimulation (rTMS) has been shown to be effective in reducing chronic tension-type headaches and chronic migraines (15, 16). Multifocal rTMS paradigms have also demonstrated improvements in clinical outcomes for patients with episodic migraine by reducing the number of migraine days (17). Given this encouraging evidence, a consensus panel has recently provided a strong recommendation for using rTMS for migraines (18).

Overall, these findings emphasize that rTMS can both induce and alleviate headaches, though the specific stimulation parameters and patient characteristics which determine these outcomes remain unclear. The seeming contrast highlights a gap in our understanding of the factors underlying rTMS-induced headaches and their clinical implications.

The current study aims to fill this gap by identifying associations between rTMS-induced headaches and technical parameters such as motor threshold, stimulation intensity, stimulation frequency, and their interaction with patient-specific characteristics such as severity of depression, biological sex, and age. We defined rTMS-induced headaches as treatment-emergent headaches that occur after the start of rTMS treatment and persist beyond the stimulation session. We differentiated headaches from scalp discomfort or pain during the stimulation. Our central hypotheses were that a) the stimulation intensity plays a role in the occurrence of headaches; and b) headaches as a side effect will have a minimal to moderate negative impact on the final clinical outcomes.

## 2 Methods

### 2.1 Subjects

This study is a retrospective chart review of data from 938 individuals who received repetitive Transcranial Magnetic Stimulation (rTMS) treatment for MDD between April 2017, and September 2023. Each patient underwent a course of at least 30 rTMS treatment sessions. Given that this a retrospective chart review, we obtained IRB approval to examine the anonymized files. Human Ethics and Consent to Participate declarations: not applicable. All performed procedures were in accordance with the ethical standards of the Declaration of Helsinki and were approved by the UCLA Medical Institutional Review Board 3, IRB # IRB-23-1934. To minimize confounding factors, patients with chronic fatigue syndrome, fibromyalgia, or obsessive-compulsive disorder were excluded from the analysis, as these conditions can be associated with chronic pain and headache disorders.

### 2.2 Headache definition and classification

To examine the relationship between rTMS and headache occurrence, patients were categorized into two groups: Headache (HA) and No-Headache (noHA), based on clinical notes documented during the first 15 treatment sessions. The focus on the first 15 sessions was chosen because the first occurrence of headaches typically happens early in the treatment course, usually within the first five to ten sessions (19).

To perform this categorization, TMS physicians performed a systematic documentation of treatment-related side effects, including headaches at every visit, which was recorded in clinical notes. Physicians differentiated between headaches existing only prior to rTMS treatment (excluded from anlayses), scalp pain and discomfort during rTMS stimulation (excluded from anlayses), and treatment-emergent headaches that outlasted the session. For the purpose of this study, “headaches” were defined as any subjective report of moderate to severe new-onset or exacerbated headaches, which outlasted the TMS session by multiple hours or which started later on the same day following rTMS treatment. Reports of headaches that occurred during the session, but did not persist after rTMS offset, were not included. Headaches could be located anywhere on the head. Reports of transient scalp pain or discomfort during stimulation were not counted as headaches. Patients were assigned to the HA group, irrespectively of whether they had a single or repeated occurrence of rTMS-induced headaches over multiple sessions. This binarized coding approach allowed for a quantitative analysis of how the presence or absence of treatment-emergent headaches affected clinical outcomes over the course of treatment.

It is important to note that the UCLA TMS clinic has a clearly formulated management plan for treatment-emergent headaches. It includes the adjustment of stimulation parameters including the reduction of the stimulation intensity, the adjustment of coil rotation, and the recommendation of over-the-counter pain medicine.

### 2.3 rTMS treatment procedures

All patients received rTMS therapy following a standardized clinical protocol using one of three stimulation devices: Magstim, MagVenture, or NeuroStar. Given different units of stimulation intensity across devices, we classified each patient based on the device that was used in the first ten session, which is when headaches initially occur, even if some patients did not receive treatment on the same stimulation device throughout the entirety of their treatment course. Using this approach, 510 patients initiated their treatment with MagVenture, 348 with Magstim, and 80 with NeuroStar.

Before treatment, each patient’s resting motor threshold (MT) was determined by identifying the minimum stimulation intensity required to elicit a visible motor response in the abductor pollicis brevis in at least 5 of 10 trials. All patients underwent treatment initially with either 10 Hz (80% of patients) or iTBS (20% of patients) to the left DLPFC. Clinicians adjusted stimulation intensity, coil angle, and the number of pulses administered as needed to manage patient comfort and intensity was increased as tolerated to reach the target stimulation intensity of 120% MT to maximize therapeutic benefit, as per our previously reported measurement-based care algorithm (20). All rTMS session were administered using a conventional treatment schedule of daily treatments Monday-Friday except on holidays. We did not examine accelerated protocols. We included only patients who received non-motor areas stimulation to avoid confounds from possible analgesic effects when stimulating the motor cortex (21–23).

### 2.4 Outcome measures

To assess whether headache occurrence was associated with treatment response, depression symptom severity was measured using a widely used self-report scale, the Inventory of Depressive Symptomatology Self-Report (IDS) (24). The questionnaire was administered at baseline and every 5 treatments throughout the treatment course to monitor changes in depression symptoms over time. Treatment response was defined as 50% symptom improvement as compared to pre-treatment baseline. In addition to depression severity, demographic factors such as age and biological sex were analyzed to determine whether they influenced the likelihood of experiencing headaches or affected treatment outcomes.

### 2.5 Statistical analysis

Statistical analyses evaluating the relationship between rTMS parameters, headache occurrence, and depression outcomes were conducted in Python using SciPy version 1.11.4 and statsmodels version 0.14.0. A Chi-squared test was used to examine whether the distribution of sex differed significantly between the HA and noHA groups. Two-sample t-tests were conducted to compare the baseline depression scores and the MTs of the HA and noHA groups. Mann-Whitney U-tests were used to compare the two groups in terms of age, median stimulation intensity over the first 10 sessions, number of sessions completed in their treatment course, number of sessions required to achieve target stimulation intensity, and end of treatment depression scores due to the non-normality of the sample distributions. Tests comparing the MTs and stimulation intensities were conducted using a one-sided alternative hypothesis that the HA group would have higher values. The MTs and intensities were compared for each device type separately because different manufacturers use different units/scales. Two-proportion z-tests were conducted to compare the proportions of patients who achieved target intensity and the proportion of patients who had headaches with 10 Hz stimulation to the proportion who had headaches with iTBS stimulation. We only considered patients who had uniform treatment protocols in the first 10 sessions for our analysis comparing 10 Hz to iTBS. This criterion resulted in 118 iTBS patients and 476–10 Hz patients. To assess how the presence of rTMS-induced headaches in the first 15 sessions influenced depression symptom trajectories, a linear mixed-effects model was used with the following formula:


Raw score∼Session number *Had headaches + Sex + Age + (1∣Patient ID)

This model included session number (encoded as a categorical variable), headache occurrence, their interaction, sex, and age as fixed effects, while patient ID was modeled as a random effect to account for individual variability.

## 3 Results

### 3.1 Demographic and clinical characteristics

**Figure 1:**
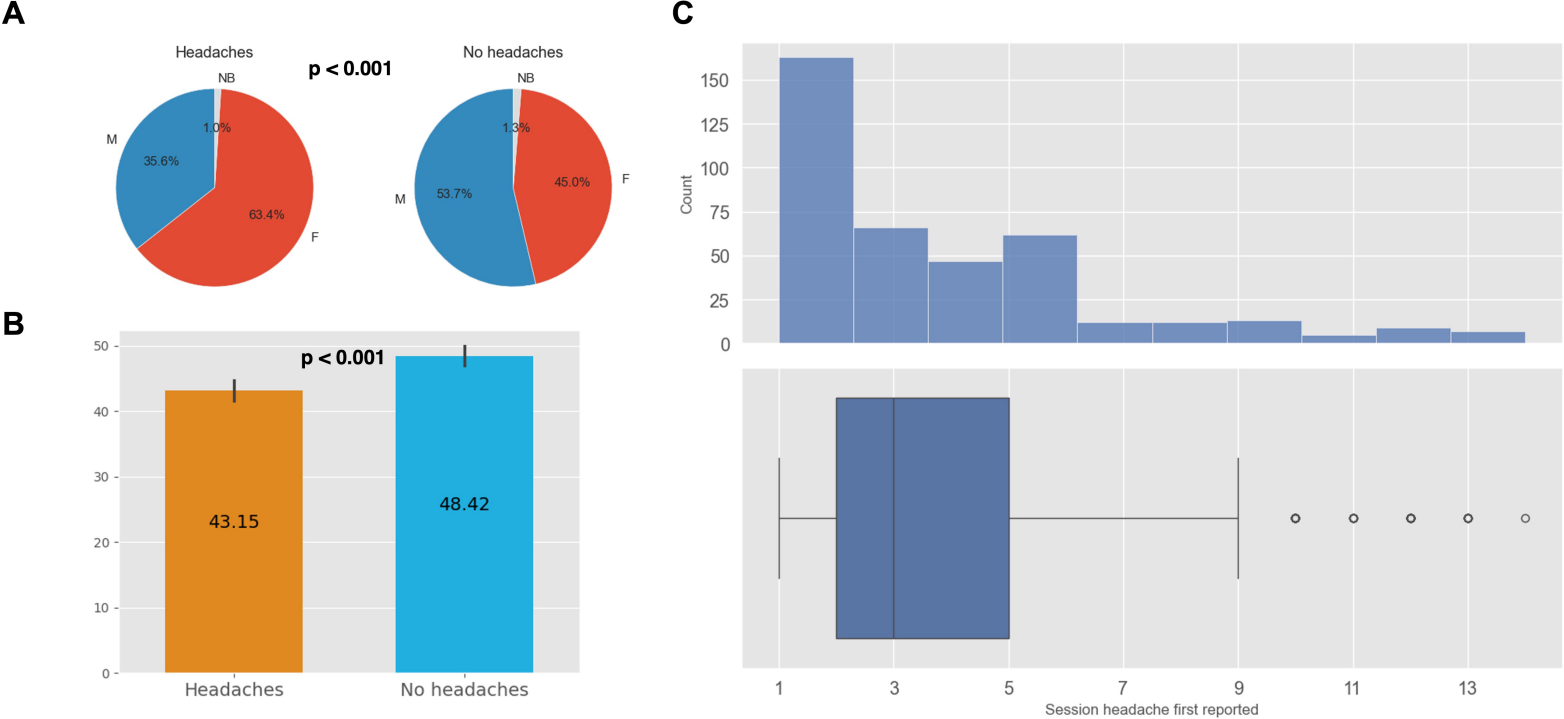
Patient demographics and headache occurrence: **(A)** Prevalence of headache occurrence by gender (HA vs. noHA groups); **(B)** Mean age of patients in HA vs. noHA groups. **(C)** Distribution of sessions numbers for first headache occurrence.

In a sample of 938 patients, 42% (n = 396) reported experiencing an rTMS-induced headache in the first 15 sessions (**Table 1**). Patients in the headache (HA) group were significantly more likely to be female (63.4%) compared to the no-headache (noHA) group (45.0%) (χ^2^ = 31.03, p = 9.25 x 10^-6^) (**Figure 1A**). Additionally, HA patients were significantly younger than noHA patients, with a mean age of 43.15 years compared to 48.42 years, respectively (U = 87055, p = 2.16 × 10^-6^; see **Table 1**, **Figure 1B**) and a posthoc correlation analysis revealed a significant negative correlation between age and depression symptom severity (r = -0.21, p < 0.01). In line with previous reports, headaches occurred early in the treatment course (median session # 3, 75^th^ percentile reached at session #5, **Figure 1C**). Only 3% of the patients in the headache group indicated a history of headaches that were further aggravated by rTMS. 97% of patients did not report pre-existing headaches.

**Table 1:** Demographic and clinical characteristics of patients with and without rTMS-induced headaches.

Variable	Headaches (n = 396) (mean ± std)	No headaches (n = 542)	Test-statistic
Male/Female/Non-Binary	141/251/4	291/244/7	Chi-squared test: χ^2^ = 31.03, p = 9.25 x 10^-6^
Age	43.15 ± 16.44	48.42 ± 17.16	Mann Whitney U test: U = 87055, p = 2.16 x 10^-6^
Baseline IDS	45.37 ± 10.96	41.72 ± 11.10	Two-sample t-test: t = 4.81, p = 1.79 x 10^-6^
# of rTMS sessions (maximum of 35)	26.10 ± 8.63	23.88 ± 10.53	Mann-Whitney U test: u = 11449, p = 0.28
Stimulator type	Magstim: 151 MagVenture: 216 Neurostar: 29	Magstim: 197 MagVenture: 294 Neurostar: 51	
% of patients who Reached target stimulation intensity (120% MT)	82.1%	83.5%	Two-proportion z-test: z = -0.559, p = 0.58
# of sessions to reach target stimulation intensity	6.20 ± 6.74	5.82 ± 6.86	Mann-Whitney U test: U = 72047.5, p = 0.02
Stimulation protocol (% with protocol who had/didn’t have headaches)	iTBS: 43.2% 10Hz: 46.0%	iTBS: 56.8% 10Hz: 54.0%	Two-proportion z-test (proportion HA 10 Hz vs. proportion HA iTBS): z = 0.54, p = 0.59
Final IDS/Raw Scores at end of treatment	IDS: 29.50 ± 15.51	IDS: 27.02 ± 13.90	Mann-Whitney U test IDS: U = 84153.5, p = 0.04
End of treatment percent improvement	IDS: 35.5%% ± 29.9%	IDS: 36.8% ± 28.9%	Two-sample t-test, IDS: t = -0.59, p = 0.56

### 3.2 TMS stimulation parameters

**Figure 2:**
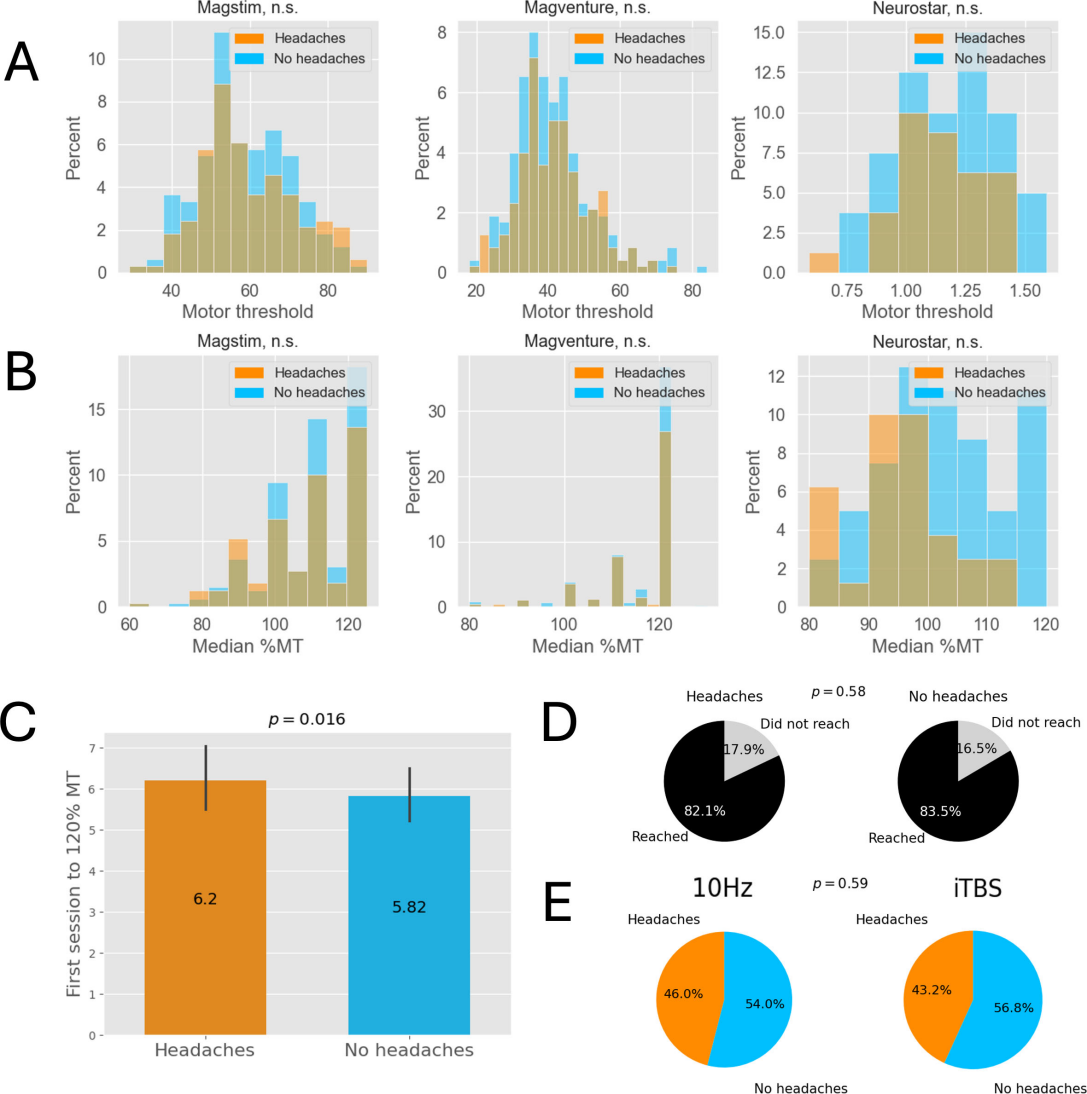
Stimulation parameters by group. **(A)** Distribution of Motor Thresholds by TMS stimulator types and HA status. **(B)** Median stimulation intensity (%MT) over the first ten sessions in each group. **(C)** Number of sessions required to reach target stimulation intensity (120% MT). **(D)** Proportion of patients who reached 120% MT. **(E)** Proportion of patients experiencing headaches in 10Hz vs. iTBS stimulation protocols.

There were no significant differences between the HA and noHA groups regarding motor threshold, or median stimulation intensity in the first 10 sessions. The number of sessions required to reach the target stimulation intensity was significantly greater in the HA than in the noHA group (U = 72047, p = 0.016), though the proportion of participants who eventually reached the target stimulation intensity was not significantly different (z = -0.559, p = 0.58). The proportion of patients with headaches who received 10Hz (46.0%) or iTBS (43.2%) was not significantly different (z = 0.54, p = 0.59) (**Table 1**, **Figures 2A–E**).

### 3.3 Baseline clinical severity, treatment outcomes and completion rates

**Figure 3:**
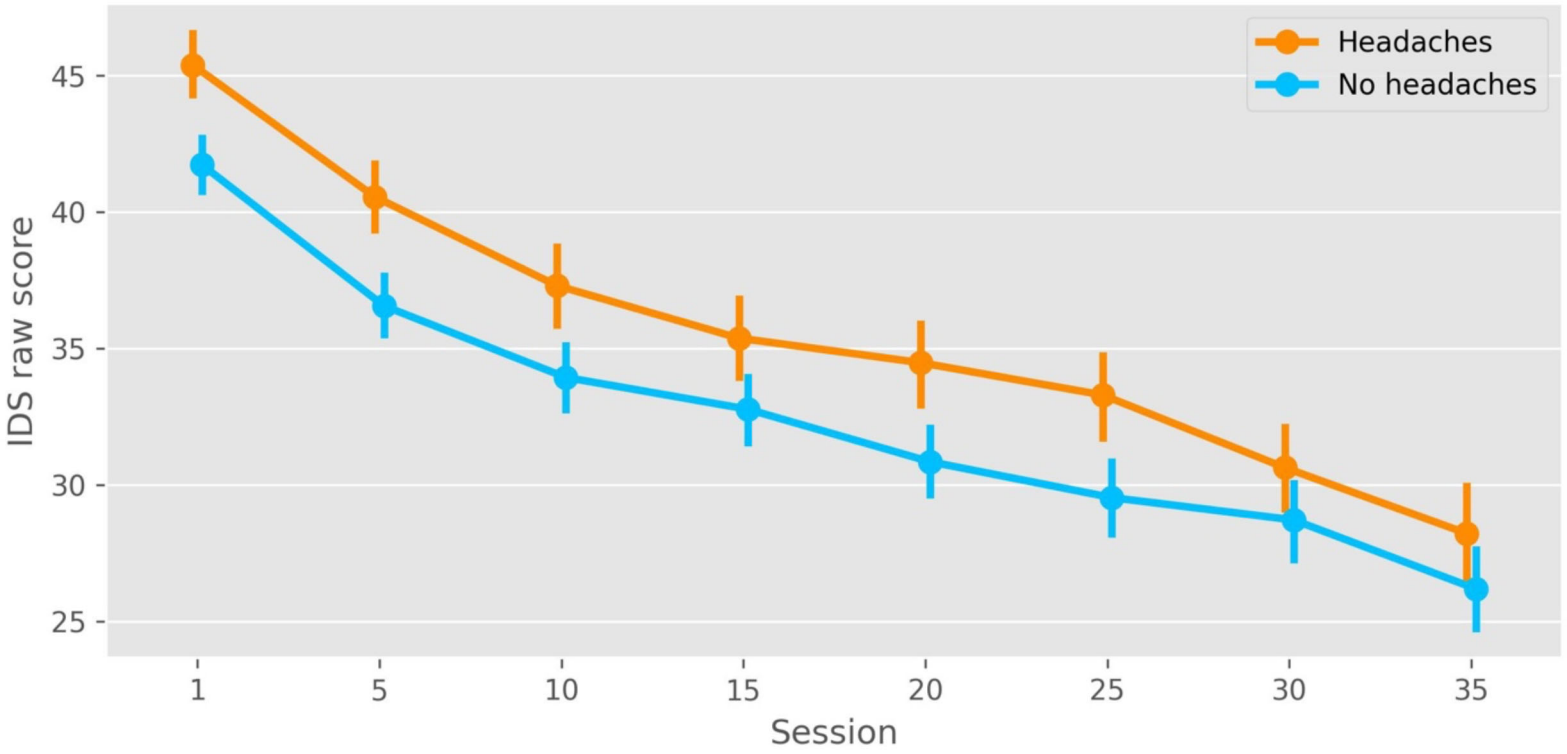
Clinical score trajectories for HA vs. noHA groups. Linear mixed-effects model showed a significant main effect of group (HA vs noHA), session number (score decrease over time) and also a group x session interaction.

The linear mixed-effects model showed a significant main effect of the presence of headaches on the IDS (β = 3.09, p = 0.0008), indicating that patients who had headaches had overall significantly higher scores throughout their treatment course. The main effects of age and sex were both significant factors, with younger patients and females exhibiting higher depression severity (β = -0.051, p = 0.03 (age), β = 2.74, p = 0.001 (gender)). Session number was strongly associated with symptom reduction (p < 10^-22^), confirming that rTMS was effective at reducing depression symptoms. There was a significant interaction between session number and the presence of headache at session 30 (β = -1.4, p = 0.043), but not at other timepoints. This suggests that the large difference in scores observed at baseline was maintained until session 30, with a convergence in the raw scores between the HA and noHA groups toward the end of treatment (**Figure 3A**). *Post-hoc* t-tests comparing percent improvement between the HA and noHA groups did not indicate a significant difference at treatment 30. While the total average number of completed rTMS sessions was not significantly different between the groups (**Table 1**), there was a significant difference in dropout rates between the HA and noHA groups. Counterintuitively, the noHA groups had *more* dropouts (4% HA group vs. 10.7% noHA group, see discussion).

## 4 Discussion

This study provides data on the prevalence and impact of rTMS-induced headaches in patients undergoing treatment for MDD. Treatment emergent headaches were reported by 42% of patients, particularly among female and younger patients, yet this side effect did not significantly affect the clinical response rates or impact the adherence to the treatment schedule. Against expectations, we did not find any technical parameters of rTMS protocols (MT, stimulation intensity, or protocol type) to be associated with headache occurrence.

Our findings corroborate prior reports indicating that treatment-emergent headaches are a common side effect of rTMS (8, 12). The observed demographic patterns also reflect broader trends in the literature where female patients have a higher prevalence of chronic pain and are more susceptible to depression and its somatic manifestations (25). The fact that younger patients, however, presented with higher levels of rTMS-induced headaches is somewhat surprising and has not been reported previously. In our sample, younger individuals had the most severe depressive symptoms, and more severely depressed individuals also had the highest likelihood of experiencing rTMS-related headaches. We thus propose that younger age might coincide with generally more severe depression, which in turn is a predisposition for higher pain sensitivity. This is consistent with prior studies showing that younger adults not only have a rising prevalence of experiencing severe anxiety, depression and pain (26, 27), but also have with greater symptom burden and functional impairment (28, 29). Possible factors for this youth mental health crisis might be convergent socio-economic “megatrends,” including intergenerational inequality, employment instability, the impact of unregulated social media and age-related disparity in resilience (30). While younger adults exhibit higher levels of catastrophizing, older adults report superior pain acceptance and self-efficacy (31). We suggest that the age and sex-related findings are thus not specific to rTMS treatment but are possibly representative of both depression and chronic pain patient populations in general.

Our data did not reveal any statistically significant differences in technical parameters between the HA and noHA groups. Motor threshold (MT), stimulation intensity and protocol type (10 Hz vs. iTBS) showed no meaningful associations with headache incidence. This observation is in line with the report by Wang et al. (8) who reported that variations in motor threshold and total pulses per session did not significantly impact the occurrence of headaches. This may indicate that technical factors may not be the primary contributors to headaches as an rTMS treatment-emergent side effect. This finding is important, as it challenges assumptions that technical aspects of rTMS delivery are primary drivers of rTMS-induced headaches. Instead, our data point toward internal patient factors—possibly psychological or physiological—as more likely contributors.

In the context of treatment outcomes, headache incidence did not significantly correlate with worse outcomes in depression measured by session 30 or 35. This suggests that patients can receive substantial treatment benefits, even if they experience headaches early in the treatment course. Interestingly, the group without headaches had a significantly higher dropout rate (10.7%) than the headaches group (4.0%). Although initially counterintuitive, this finding may be explained by a subjectively perceived lower treatment burden: the headache group needed significantly more time to reach target stimulation intensity which might have overall increased the tolerability of treatment. Furthermore, patients in the headache group were systematically encouraged to use analgesics to manage pain. This proactive pain management, combined with a more gradual titration schedule, likely reduced the overall treatment burden for the headache group, potentially explaining their higher retention rate compared to the more rapid ramping schedule experienced by the no headache group.

While it remains unclear why exactly rTMS to non-motor areas causes persistant headaches, they likely arise from the sensitization of the trigeminovascular system (5, 8, 32). Repetitive magnetic pulses stimulate superficial branches of the trigeminal nerve, sending afferent signals to the trigeminocervical complex (TCC). This may trigger the release of vasoactive neuropeptides, such as CGRP, leading to intracranial vasodilation and “sterile inflammation” of the meninges (33). Furthermore, “forced posture” and head immobilization during treatment (34) create sustained cervical strain. Due to the anatomical convergence of cervical and trigeminal inputs within the TCC, this strain is often perceived as a referred cervicogenic headache. The effectiveness of oral analgesics (5) suggests these headaches are mediated by traditional inflammatory pathways rather than direct cortical trauma.

Combined these observations suggest that patients’ overall treatment experience and complaints should be taken seriously to enhance clinical outcomes. In our practice, rTMS physicians paid close attention to the report of treatment-emergent headaches and immediately addressed any concerns by reducing stimulation intensity, adjusting coil rotation, or by recommending over-the-counter pain medicine. Given that headache occurrence during the first three weeks of treatment didn’t reduce eventual depression outcome, these interventions taken by our clinicians, or other interventions to reduce headache severity can be a valuable component of clinical care during a course of rTMS for depression. We argue that the prompt management of headaches is important to ensure treatment adherence, also in patients who do not spontaneously report headaches or other side-effects. We recommend that clinicians should make efforts to improve patient comfort through consulting and reassurance, pain management strategies, or slight protocol modifications when appropriate.

In summary, while technical parameters of rTMS do not appear to influence headache incidence, patient characteristics such as depression severity, sex, and age play a significant role in the occurrence of headaches.

### 4.1 Limitations

This study relied on retrospective chart review, which may have introduced variability in how headache reports were documented by different clinicians. The absence of standardized pain assessments limits the ability to quantify headache severity or distinguish between different types of headaches. Accompanying symptoms such as nausea or sensitivity to light or the frequency of headaches were not systematically collected. We also did not assess potential psychological predictors of pain, such as catastrophizing, which could be important mediators. We further acknowledge that the use of multiple different rTMS devices across the patient cohort restricted our ability to compare the impact of device-specific parameters on headache incidence. Because different rTMS machines vary in their magnetic field geometry, coil design, and pulse characteristics—factors known to influence the depth and intensity of cortical stimulation—the lack of hardware standardization may introduce confounding variables. Future research utilizing standardized hardware platforms is warranted to isolate the precise relationship between specific stimulator and patient-reported headaches. Finally, the influence of concurrent medications or pre-existing chronic pain conditions was not accounted for, which could have impacted headache reports and treatment outcomes.

## 5 Conclusions

Despite their prevalence, rTMS-induced headaches did not negatively affect overall treatment outcomes, suggesting that patients can benefit from rTMS treatment despite the experience of treatment-emergent headaches early in the treatment course. Our findings indicate that internal factors such as age, sex, and clinical severity play a larger role in headache incidence than technical stimulation parameters. We suggest that addressing the occurrence of headaches early in the treatment course may improve the overall patient experience. Future work should explore personalized strategies to better support patients prone to side effects during rTMS.

## Data Availability

The datasets generated and analyzed during the current study are not publicly available due to participant confidentiality and ethical restrictions but are available from the corresponding author on reasonable request.
